# Brain-Specific Serine-47 Modification of Cytochrome *c* Regulates Cytochrome *c* Oxidase Activity Attenuating ROS Production and Cell Death: Implications for Ischemia/Reperfusion Injury and Akt Signaling

**DOI:** 10.3390/cells9081843

**Published:** 2020-08-06

**Authors:** Hasini A. Kalpage, Junmei Wan, Paul T. Morse, Icksoo Lee, Maik Hüttemann

**Affiliations:** 1 Center for Molecular Medicine and Genetics, Wayne State University, Detroit, MI 48201, USA; hkalpage@med.wayne.edu (H.A.K.); am4472@wayne.edu (J.W.); morsepa@wayne.edu (P.T.M.); 2 College of Medicine, Dankook University, Cheonan-si, Chungcheongnam-do 31116, Korea; icksoolee@dankook.ac.kr; 3 Department of Biochemistry, Microbiology and Immunology, Wayne State University, Detroit, MI 48201, USA

**Keywords:** cytochrome *c*, phosphorylation, ischemia/reperfusion injury, cell signaling, apoptosis, electron transport chain, brain, reactive oxygen species, mitochondrial membrane potential

## Abstract

We previously reported that serine-47 (S47) phosphorylation of cytochrome *c* (Cyt*c*) in the brain results in lower cytochrome *c* oxidase (COX) activity and caspase-3 activity in vitro. We here analyze the effect of S47 modification in fibroblast cell lines stably expressing S47E phosphomimetic Cyt*c*, unphosphorylated WT, or S47A Cyt*c*. Our results show that S47E Cyt*c* results in partial inhibition of mitochondrial respiration corresponding with lower mitochondrial membrane potentials (ΔΨ_m_) and reduced reactive oxygen species (ROS) production. When exposed to an oxygen-glucose deprivation/reoxygenation (OGD/R) model simulating ischemia/reperfusion injury, the Cyt*c* S47E phosphomimetic cell line showed minimal ROS generation compared to the unphosphorylated WT Cyt*c* cell line that generated high levels of ROS upon reoxygenation. Consequently, the S47E Cyt*c* cell line also resulted in significantly lower cell death upon exposure to OGD/R, confirming the cytoprotective role of S47 phosphorylation of Cyt*c*. S47E Cyt*c* also resulted in lower cell death upon H_2_O_2_ treatment. Finally, we propose that pro-survival kinase Akt (protein kinase B) is a likely mediator of the S47 phosphorylation of Cyt*c* in the brain. Akt inhibitor wortmannin abolished S47 phosphorylation of Cyt*c*, while the Akt activator SC79 maintained S47 phosphorylation of Cyt*c*. Overall, our results suggest that loss of S47 phosphorylation of Cyt*c* during brain ischemia drives reperfusion injury through maximal electron transport chain flux, ΔΨ_m_ hyperpolarization, and ROS-triggered cell death.

## 1. Introduction

Cytochrome *c* (Cyt*c*) is a small, 104 amino acid protein, with a covalently attached heme group. It functions at the intersection between cellular respiration and intrinsic apoptosis, linking the two pathways. Cyt*c* plays a role in cellular respiration by functioning as the electron carrier between complex III and complex IV (cytochrome *c* oxidase, COX) in the mitochondrial electron transport chain (ETC) [1,2]. In contrast, the release of Cyt*c* from the mitochondria into the cytosol is considered the committing step for intrinsic apoptosis. Cyt*c* acts as a trigger of apoptosis by interacting with apoptosis protease activating factor-1 (Apaf-1) to form the apoptosome, which activates caspase-9 to initiate the caspase cascade, the executioner pathway of intrinsic apoptosis [3,4]. Cyt*c* serves as a key metabolic regulator and orchestrator of cellular life and death decisions. In addition to respiration and apoptosis, Cyt*c* has multiple other cellular functions, such as reactive oxygen species (ROS) scavenging, cardiolipin peroxidase activity, redox-coupled protein import, and ROS formation via the p66Shc protein [1,5]. The COX-catalyzed electron transfer from Cyt*c* to oxygen is the proposed rate-limiting step of the ETC [2,6,7]. Therefore, the functions of Cyt*c* are tightly regulated by all major regulatory mechanisms, such as allosteric regulation by ATP, expression of tissue-specific isoforms, and reversible post-translational modifications (PTMs), of which phosphorylations are physiologically most relevant [5].

So far, five tissue-specific phosphorylations have been mapped on mammalian Cyt*c*—Y97 [8,9], Y48 [10], T28 [11], S47 [12], and T58 [13]. These modifications strongly impact the functions of the protein, including respiration, apoptosis, cardiolipin peroxidase activity, and ROS scavenging capability [11,13,14]. We recently showed that S47 phosphorylation is the basal PTM found in the mammalian brain. This phosphorylation was found in 30% of the Cyt*c* pool, making it a biologically significant modification. Furthermore, this phosphorylation was found to be completely lost under ischemia, implying a critical role for this modification in the brain under healthy basal conditions. Our initial study used in vivo phosphorylated and S47E phosphomimetic Cyt*c* for the in vitro functional characterization with consistent results between the two systems, demonstrating that our phosphomimetic replacement serves as a good model. We reported that brain COX activity was reduced by 54.3%, caspase-3 activity was reduced by 64.5%, and cardiolipin peroxidase activity was reduced by about 50% in the presence of the S47 phosphomimetic S47E compared to unphosphorylated WT Cyt*c* [12]. These experiments were performed in a cell-free in vitro system using purified recombinant proteins. Given the pathological relevance of this modification under ischemia, a condition observed in stroke and global brain ischemia following cardiac arrest, we further extended characterization of S47 phosphorylation into a live cell culture model with exposure to oxygen-glucose deprivation/reoxygenation (OGD/R) mimicking ischemia/reperfusion injury that takes place as a result of stroke [15].

Globally, strokes are the second leading cause of death and the third leading cause of disability [16]. Most strokes are ischemic in origin, where blood flow to the brain is disrupted by occlusion of a blood vessel. Restoration of blood flow further damages the ischemic core and surrounding tissue called the penumbra as a result of reperfusion injury, exacerbating the stroke pathology [17]. ROS are implicated in reperfusion injury [18], and since the mitochondrial ETC is the primary source of cellular ROS, the ETC thus plays an important role in ischemia/reperfusion injury [19]. We propose that the regulatory functions of S47 phosphorylation of Cyt*c*, which are lost during ischemia, play a central role in brain ischemia/reperfusion injury through the following mechanism: Under ischemic stress calcium builds up in the mitochondria, leading to dephosphorylation of Cyt*c* and other ETC proteins. This causes hyperactivation of the mitochondrial ETC during reperfusion when oxygen becomes available again, resulting in pathologically high mitochondrial membrane potentials (ΔΨ_m_). In turn, this triggers an exponential increase in the generation of ROS, which causes further tissue damage and initiates cell death cascades [17]. Our data support the concept that cell death due to ischemia/reperfusion injury takes place via a Cyt*c*-centered mechanism mediated by loss of its phosphorylations. Furthermore, we propose that S47 phosphorylation is likely mediated by the pro-survival kinase Akt under normal conditions.

## 2. Materials and Methods

### 2.1. Cell Culture and Stable Transfection of Cytc Constructs

WT Cyt*c* cloned into pBABE-puro expression plasmid (Addgene, Cambridge, MA, USA) was used to generate S47A and S47E Cyt*c* variants using site-directed mutagenesis (Agilent Technologies, Santa Clara, CA, USA), as previously described [12], with the following mutagenesis oligonucleotides: S47E forward primer: 5′-CCAGGCTGCTGGATTCGAGTACACAGATGCC-3′, S47E reverse primer: 5′-GGCATCTGTGTACTCGAATCCAGCAGCCTGG-3′, S47A forward primer: 5′-CCAGGCTGCTGGATTCGCTTACACAGATGCC-3′, S47A reverse primer: 5′-GGCATCTGTGTAAGCGAATCCAGCAGCCTGG-3′. WT, S47E, and S47A Cyt*c* expression constructs were stably transfected into Cyt*c* double-knockout mouse lung fibroblasts (a kind gift from Dr. Carlos Moraes, University of Miami, Coral Gables, FL, USA) using Transfast transfection reagent (Promega, Madison, WI, USA) in a 1:1 transfection reagent to DNA ratio, as described in the manufacturer’s protocol. The transfected cells were cultured in DMEM supplemented with 10% FBS (Sigma-Aldrich, St. Louis, MO, USA), 100 µg/mL primocin (Invivogen, San Diego, CA, USA), 1 mM sodium pyruvate, and 50 mg/mL uridine in selection media with 3 µg/mL puromycin at 37 °C in 5% CO_2_.

### 2.2. Gel Electrophoresis and Western Blotting

Cyt*c* expression in stably-transfected Cyt*c* double-knockout mouse lung fibroblasts was confirmed with a mouse Cyt*c* antibody (BD pharmingen, San Jose, CA, USA, #556433) used at a 1:1000 dilution in 5% dry milk. Briefly, cells were lysed in RIPA lysis buffer supplemented with protease inhibitor cocktail (Sigma-Aldrich). The cellular protein lysates were quantified using the DC protein assay (Bio-Rad, Hercules, CA, USA) based on the Lowry method. Thirty micrograms of total cell lysate of each cell line were run on a 10% tris-tricine SDS-PAGE gel in the presence of anode (200 mM Tris, pH 8.9) and cathode buffers (100 mM Tris, 100 mM Tricine, 0.1% SDS, pH 8.25). The gel was transferred onto a PVDF membrane (Bio-Rad) using a semi-dry apparatus (Bio-Rad) and incubated with the primary antibody overnight at 4 °C. The same lysates were also probed for subunits of the ETC complexes with the following antibodies at a dilution of 1:1000: NDUFB6 (NADH:Ubiquinone Oxidoreductase Subunit B6) for complex I (Abcam, Cambridge, MA, USA #ab110244), SDHA (Succinate Dehydrogenase Complex Flavoprotein Subunit A) for complex II (Abcam, #ab14715), UQCRC1 (Ubiquinol-Cytochrome *c* Reductase Core Protein 1) for complex III (Abcam, #ab110252), COX4 (Cytochrome *c* Oxidase Subunit 4) for complex IV (Proteintech, Rosemont, IL, USA, #11242-1-AP), and ATP5A (ATP Synthase Subunit Alpha) for complex V (Abcam, #ab110273). Cell lysates were also probed with a 4-hydroxynonenal monoclonal antibody (clone 12F7) (Thermoscientific, Waltham, MA, USA, #MA5-27570). The blots were probed for tubulin (Proteintech, #11224-1-AP) as a loading control. HRP-conjugated mouse and rabbit secondary antibodies (GE Healthcare, Chicago, IL, USA) were used at a 1:5000 dilution. The blots were visualized using the HyGLO chemiluminescent HRP detection reagent (Denville Scientific Inc, Metuchen, NJ, USA).

### 2.3. Measurement of Oxygen Consumption and Extracellular Acidification Rate

Cells were seeded at a density of 25,000/well in a gelatin-coated XF24 plate (Agilent, #100777-004). After overnight incubation, the growth medium was replaced with 675 µL of Seahorse media (#D5030 DMEM) supplemented with 10 mM galactose without FBS or phenol red. In independent experiments, the growth medium was also replaced with 675 μL of seahorse media supplemented with 10 mM sodium pyruvate and 10 mM glucose. The cells were incubated in a CO_2_-free incubator for 1 h, and the basal oxygen consumption rate (OCR) was measured, followed by mitochondrial stress test with sequential injections of oligomycin (1 μM), trifluoromethoxy carbonylcyanide phenylhydrazone (FCCP, 0.5 μM), and rotenone/antimycin A (1 μM) in an XFe24 Seahorse bioanalyzer (Seahorse Biosciences, North Billerica, MA, USA), according to the manufacturer’s protocol. Extracellular acidification rate (ECAR) was determined as a measure of the rate of glycolysis in Seahorse medium supplemented with 10 mM sodium pyruvate and 10 mM glucose.

### 2.4. Measurement of Membrane Potential

Mitochondrial membrane potential (ΔΨ_m_) was measured using the ratiometric JC-1 (5,5′,6,6′-tetrachloro-1,1′,3,3′-tetraethylbenzimidazolylcarbocyanine iodide) probe (Invitrogen, Carlsbad, CA, USA, #T3168). Cells were seeded (25,000 cells/well) in a black 96-well plate (Corning, #CLS3603). The growth medium was changed into FBS-free, phenol red-free high glucose (4.5 g/L) DMEM cell culture medium supplemented with 0.5 µM JC-1 and incubated for 30 min. Cells were washed with 1× PBS twice. Green fluorescence (excitation 485 nm/emission 527 nm) and red fluorescence (excitation 485 nm/emission 590 nm) of the cells were measured using a Fluorskan Ascent FL plate reader (Thermoscientific). ΔΨ_m_ was represented as a ratio of red/green fluorescence. ΔΨ_m_ uncoupler FCCP (1 µM) was used as a negative control.

### 2.5. Measurement of Mitochondrial ROS Production

Mitochondrial ROS production was measured using MitoSOX red mitochondrial superoxide indicator probe (Invitrogen, #M36008). Cells were seeded (100,000 cells/well) on a 24-well plate and incubated in FBS-free, phenol red-free high glucose (4.5 g/L) DMEM cell culture medium supplemented with 5 µM MitoSOX for 30 min at 37 °C. Cells were washed with 1× PBS. Fluorescence (excitation 510 nm/emission 580 nm) was measured using a Synergy H1 plate reader (BioTek, Winooski, VT, USA).

### 2.6. Measurement of ATP Levels

Cultured fibroblasts were collected by scraping. The pellets were immediately frozen in less than 90 s and stored at −80 °C until analysis. The samples were boiled in the presence of 300 µL of boiling buffer (100 mM Tris-Cl, 4 mM EDTA, pH 7.75) and sonicated on ice. The sample lysates were diluted 300-fold. The ATP concentration of 40 µL diluted lysate was measured using the ATP bioluminescence assay kit HS II (Roche, Indianapolis, IN, USA), according to the manufacturer’s protocol, using an Optocomp 1 luminometer (MGM Instruments, Hamden, CT, USA). The results were normalized to the protein concentration determined using the DC protein assay kit (Bio-Rad).

### 2.7. Oxygen-Glucose Deprivation/Reoxygenation (OGD/R) Experiments

To model ischemia/reperfusion conditions, stable cell lines expressing Cyt*c* variants were exposed to transient oxygen-glucose deprivation (OGD). Cells were seeded and cultured according to our standard protocol described above. The media was exchanged into glucose-free, FBS-free, phenol red-free DMEM (Gibco, #A1443001) bubbled with 95% N_2_ and 5% CO_2_ (ischemia-mimetic media). Cells were incubated in ischemia-mimetic media with a 1% O_2_ and 5% CO_2_ atmosphere in a hypoxic chamber under the control of ProOx 110 oxygen and ProCO_2_ carbon dioxide controllers (Biospehrix, Redfield, NY, USA) for 90 min at 37 °C unless otherwise stated. After ischemia, cells were reoxygenated for 30 min with glucose-containing, FBS-free, phenol red-free DMEM culture medium (Gibco, #31053028) supplemented with the respective probe.

### 2.8. Measurement of Cell Death Using Annexin V/PI Staining

Cells were exposed to OGD/R (12 h ischemia/1 h reperfusion), as previously described [20], or treated with H_2_O_2_ (400 µM for 16 h). Cells were harvested, washed with cold 1× PBS twice, and resuspended in 1× annexin V binding buffer (0.1 M HEPES, pH 7.4, 1.4 M NaCl, and 25 mM CaCl_2_). A total of 1 × 10^6^ cells were stained with annexin V and propidium iodide (PI), as described in the annexin V/PI staining apoptosis kit manual (BD biosciences, San Jose, CA, USA). The cells were resuspended in 1 mL of annexin V binding buffer, and the data were collected using a flow cytometer (FloMax, Sysmex America, Inc., Lincolnshire, IL, USA). The results were analyzed using FCS Express 7 software (De Novo Software, Glendale, CA, USA).

### 2.9. Akt Kinase Assay

Recombinant Akt 1 (#A16-10G), Akt 2 (#A17-10G), and Akt 3 (#A18-10G) isoforms were purchased from SignalChem (Richmond, British Columbia, Canada). The in vitro kinase reaction was performed in 50 mM HEPES (pH 7.5), 10 mM MgCl_2_, 1 mM EGTA, 200 µM ATP, 0.01% Brij-35 buffer with 2 µM Cyt*c* at 30 °C. The serine phosphorylation state of Cyt*c* was determined by Western blotting at times 0, 15, and 30 min. The blots were probed with a 1:5000 dilution of phosphoserine antibody (Millipore, Burlington, MA, USA #AB1603) in 5% BSA-TBST buffer, followed by a 1:10,000 dilution of HRP-conjugated rabbit secondary antibody (GE Healthcare).

### 2.10. Tissue Treatment with Pharmacological Compounds and Cytochrome c Purification

Freshly harvested pig brain tissue was thoroughly washed with ice-cold 1× PBS, cut into smaller pieces, and minced using a commercial meat grinder. All procedures involving animal tissues were approved by the Wayne State University Institutional Animal Care and Use Committee. The tissue was suspended in 250 mM sucrose, 20 mM Tris-Cl buffer supplemented with 10 mM potassium fluoride (KF), 2 mM ethylene glycol tetraacetic acid (EGTA), and 1 mM Phenylmethylsulfonyl fluoride (PMSF). The tissue homogenate was divided into 3 equal volumes. The fractions were treated with 13.7 µM SC79, an Akt activator (Sigma-Aldrich, #SML0749,), 10 µM wortmannin (Sigma-Aldrich, #W1628), an inhibitor of the phosphoinositide 3-kinase (PI3K)/Akt pathway, or DMSO as solvent control. Tissues were incubated at 37 °C for 30 min with stirring to allow adequate tissue respiration and oxygenation. The pH was adjusted to 7.5. Following treatment, brain Cyt*c* was purified, as previously described [12].

### 2.11. Mitochondrial Subfractionation

Freshly harvested pig brain tissue was homogenized in 250 mM sucrose, 20 mM Tris-Cl buffer supplemented with 10 mM KF, 2 mM EGTA, and 1 mM PMSF. All procedures involving animal tissues were approved by the Wayne State University Institutional Animal Care and Use Committee. The homogenate was spun at 650× *g* for 10 min to remove the tissue debris. The supernatant was filtered through a cheesecloth and spun at 14,000× *g* for 20 min to collect the mitochondria. The pellet was homogenized with a glass Dounce homogenizer and centrifuged at 300× *g* for 8 min. The supernatant was again spun at 14,000× *g* for 20 min to collect the mitochondria. The pellet was resuspended in 10 mM HEPES buffer (pH 7.5) containing 210 mM mannitol, 70 mM sucrose, and 1 mM EDTA. Mitochondrial protein content was quantified using the DC protein assay (Bio-Rad). Two sucrose solutions (1.2 M and 1.6 M) of varying density were prepared, and 16 mL of 1.6 M sucrose solution was added to a Beckmann ultra-clear tube (#344058), followed by 19 mL of 1.2 M sucrose solution. Each sucrose gradient was loaded with 5 mg of mitochondrial protein, and the tubes were spun at 27,000 rpm in a Beckmann SW28 swing rotor for 2 h at 4 °C. The mitochondrial fraction was extracted from the tube using a 5 cc syringe with an 18 gauge needle. The collected fraction was centrifuged at 50,000 rpm in a Beckmann type 75 Ti rotor. The pellet was resuspended in 250 mM sucrose, 10 mM Tris-Cl, and 20 mM EDTA buffer (pH 7.6) and spun at 22,000× *g* for 15 min. The pellet was then resuspended in a hypertonic solution (20 mM sodium phosphate, pH 7.2, 0.02% BSA) and allowed to swell for 20 min on ice, as previously described [11]. After 20 min, 1 mM ATP and 1 mM MgCl_2_ were added and further incubated for 5 min. The suspension was centrifuged at 15,000× *g* for 10 min at 4 °C. The supernatant was collected as the mitochondrial IMS (intermembrane space) fraction, and the pellet was collected as a mixture of mitoplasts, OMM (outer mitochondrial membrane), IMM (inner mitochondrial membrane), and residual IMS. The fractions were immunoblotted with the following antibodies: pAkt (CST, Danvers, MA, USA, #4060S), total-Akt (CST, #C67E7), cytochrome *c* (BD pharmingen, #556433), COX4 (Proteintech, #11242-1-AP), heat shock protein 60 (HSP60) (Proteintech, #66041), and tubulin (Proteintech, #11224-1-AP).

### 2.12. Recombinant Cytc Purification and Cytc Oxidation by Hydrogen Peroxide

WT, S47A, and S47E variants were overexpressed in C41(DE3) competent bacterial cells (Lucigen, Middleton, WI, USA) and purified using ion-exchange chromatography, as previously described [12]. WT, S47E, and S47A Cyt*c* were fully reduced with sodium dithionite and desalted on a NAP5 column (GE Healthcare). The initial rate of Cyt*c* oxidation with 100 µM H_2_O_2_ within the first 10 s was calculated, as previously described [13]. The rate of Cyt*c* oxidation is represented as % of the WT.

### 2.13. Statistical Analyses

Statistical analyses of the data were performed with MSTAT version 6.1 (University of Wisconsin, Madison, WI, USA) using the Wilcoxon rank-sum test. Data are reported as means ± SEM with three or more replicates and were considered statistically significant (*) with *p* < 0.05.

## 3. Results

### 3.1. Overexpression of WT, S47A, and S47E Cytc in Cytc Double-Knockout Cells Restores Expression of Complex I, III, and IV

**Figure 1:**
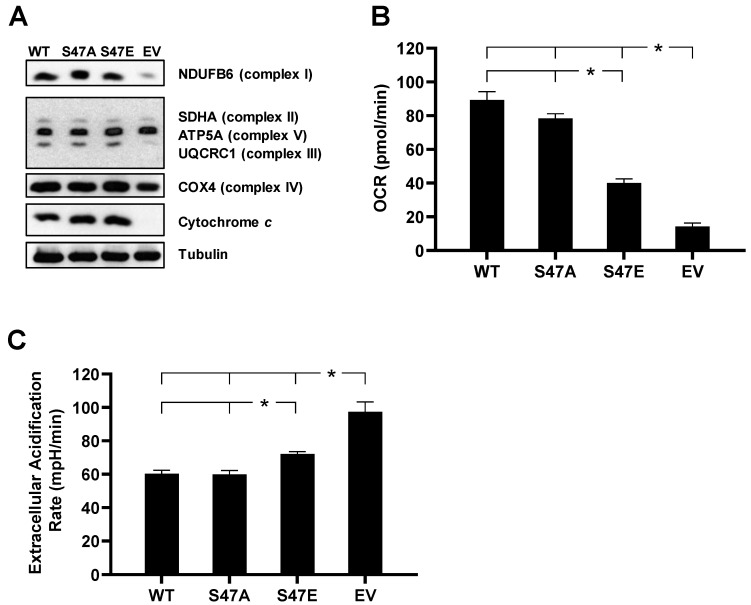
(**A**) Cytochrome c (Cyt*c*) double-knockout cells stably transfected with empty vector (EV) and wild type (WT), S47A, S47E Cyt*c* expression constructs were immunoprobed for Cyt*c*, together with loading control tubulin, and OxPhos complex subunits NDUFB6 (complex I), SDHA (complex II), UQCRC1 (complex III), COX4 (complex IV), and ATP5A (complex V). (**B**) Oxygen consumption rate (OCR) of intact cells stably expressing EV and WT, S47A, S47E Cyt*c* measured in Seahorse media supplemented with 10 mM galactose using the Seahorse bioanalyzer (*n* = 9–13). (**C**) Extracellular acidification rate (ECAR) of intact cells as a measure of the rate of glycolysis measured in Seahorse media supplemented with 10 mM glucose and 10 mM sodium pyruvate (*n* = 4–6). Data are represented as means ± SEM, * *p* < 0.05.

To study the functional effects of S47E phosphomimetic substitution of Cyt*c* in an intact cell culture system, we created cell lines stably expressing S47E Cyt*c*, WT Cyt*c*, and S47A Cyt*c* as an additional control that cannot be phosphorylated. These Cyt*c* variants were transfected into a Cyt*c* double-knockout mouse lung fibroblast cell line where both the rodent testes and somatic isoforms of Cyt*c* were knocked out [21]. Cell lines that were equally expressing WT, S47E, and S47A Cyt*c,* along with a Cyt*c*-null empty vector (EV) control, were used for the functional studies (Figure 1A). These cell lines were probed for subunits of the five complexes involved in oxidative phosphorylation (OxPhos). In the EV cell line, the protein expression levels of complex I subunit (NDUFB6), complex III subunit (UQCRC1), and complex IV subunit (COX4) were lower (Figure 1A). Consistent with previous studies [11], when Cyt*c* was expressed in Cyt*c* double-knockout cells, there was an upregulation of the ETC complexes involved in proton pumping, suggesting that reintroduced Cyt*c* was integrated into the mitochondria, resulting in OxPhos competent cells.

### 3.2. S47E Cytc Expression Results in Lower Mitochondrial Respiration and Higher Glycolysis Rates

Intact cell oxygen consumption rate of the four cell lines was measured using a Seahorse bioanalyzer. There was a 59% decrease and a 52% decrease in intact cell oxygen consumption rate (OCR) in the S47E phosphomimetic Cyt*c* cell line as compared to WT Cyt*c* cell line cultured in 10 mM galactose supplemented media (Figure 1B, Figure S1B) and 10 mM glucose and 10 mM sodium pyruvate supplemented media (Figure S2B), respectively. This was similar to the difference in OCR that was observed in the in vitro reaction of purified S47-phosphorylated Cyt*c* with purified COX analyzed in an oxygen electrode chamber [12]. The S47E cell line also resulted in lower respiration due to decreased proton leak and lower ATP-coupled respiration after the addition of oligomycin during the mitochondrial stress test (Figures S1A,C,D and S2A,C,D). The rate of glycolysis of these cells was determined indirectly by measurement of the extracellular acidification rate (ECAR). Extracellular measurement of lactic acid production is considered to be a reasonable estimate of glycolytic flux [22]. The rate of glycolysis showed a behavior opposite to that of OCR (Figure 1C, Figure S3A). The S47E cell line resulted in a significantly higher rate of extracellular acidification compared to the WT and S47A cell lines, suggesting an adaptation in cellular bioenergetics to compensate for the decrease in oxygen consumption. The Cyt*c*-null EV cell line, which is OxPhos deficient, resulted in the highest rate of glycolysis, representing a complete metabolic switch to glycolysis.

### 3.3. S47E Cytc Expressing Cells Have a Lower Mitochondrial Membrane Potential (ΔΨm) That Corresponds to Lower ROS Production

**Figure 2:**
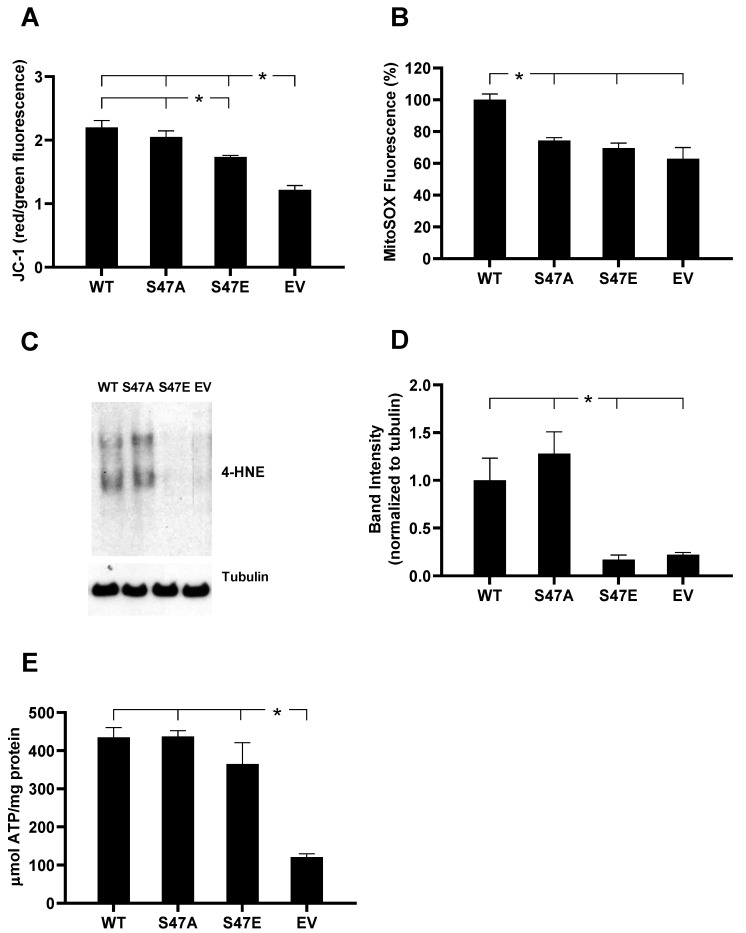
(**A**) Mitochondrial membrane potential (ΔΨ_m_) of intact cells measured using red/green fluorescence distribution of the ratiometric JC-1 probe (*n* = 6). (**B**) Mitochondrial reactive oxygen species (ROS) production in intact cells measured as % of MitoSOX fluorescence (*n* = 4). (**C**) 4-hydroxynonenal (4-HNE), a product of lipid peroxidation, immunoblotted on cells stably expressing EV and WT, S47A, S47E Cyt*c*. (**D**) Quantification of 4-HNE band density using ImageJ software (*n* = 3). (**E**) ATP levels in the cell lines stably expressing EV and WT, S47A, S47E Cyt*c* (*n* = 3). Data are represented as means ± SEM, * *p* < 0.05.

The mitochondrial membrane potential (ΔΨ_m_) in our cell lines was assessed using the ratiometric JC-1 probe. The JC-1 probe diffuses into the mitochondria and accumulates and aggregates when ΔΨ_m_ is high, resulting in red fluorescence. At low ΔΨ_m_, it exists as a monomer that emits green fluorescence. Therefore, the ratio of red/green fluorescence provides a relative measure for ΔΨ_m_. FCCP, a potent uncoupler of ΔΨ_m_, was used as a control. The phosphomimetic S47E Cytc cell line resulted in a statistically significant 21% decrease in fluorescence compared to the WT Cytc expressing cell line, indicating that the phosphomimetic replacement led to a reduction in ΔΨ_m_ (Figure 2A). It has been shown that ΔΨ_m_ is proportional to mitochondrial ROS production. However, ΔΨ_m_ and ROS do not have a linear relationship [5]. Minimal ROS levels and optimal ATP levels are maintained within a physiological, intermediate membrane potential range of about 80–120 mV [23]. When ΔΨ_m_ exceeds 140 mV, this results in an exponential increase in ROS that can be detrimental and trigger cell death [5,17]. We analyzed mitochondrial ROS production in the four cell lines using the mitochondria-specific fluorescent superoxide indicator MitoSOX. Cells expressing phosphomimetic S47E Cyt*c* showed a 30% decreased MitoSOX fluorescence, demonstrating reduced mitochondrial superoxide production compared to the WT control cell line (Figure 2B). We also probed our cell lysates for 4-HNE (4-hydroxynonenal), a product of lipid peroxidation that is an indirect readout of total cellular ROS [24]. Our results confirmed significantly lower total 4-HNE levels for the S47E phosphomimetic cell line compared to WT and S47A Cyt*c* expressing cell lines (Figure 2C,D). This finding is consistent with the direct relationship between ΔΨ_m_ and ROS production. Finally, bioluminescence produced by the ATP-dependent luciferase catalyzed luciferin oxidation reaction was used to measure cellular ATP levels. In agreement with the higher oxygen consumption rates and ΔΨ_m_ levels, both WT and S47A Cyt*c* expressing cells showed a trend towards slightly increased ATP levels compared to the S47E Cyt*c* expressing cell line (Figure 2E).

### 3.4. Phosphomimetic S47E Cytc Attenuates ROS Production upon Oxygen-Glucose Deprivation/Reoxygenation (OGD/R)

**Figure 3:**
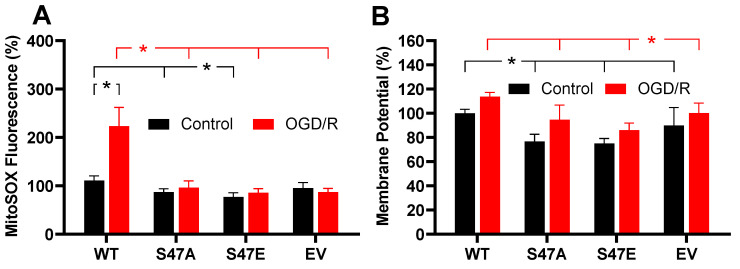
(**A**) ROS production following exposure to 90 min of oxygen-glucose deprivation, followed by 30 min of reoxygenation (OGD/R) (*n* = 9–10). (**B**) Mitochondrial membrane potential (ΔΨ_m_) determined by JC-1 fluorescence after exposure to 90 min of oxygen-glucose deprivation, followed by 30 min of reoxygenation (OGD/R) (*n* = 5–10). Black lines represent significant differences among control bars, while the red lines represent significant differences among OGD/R bars. Data are represented by means ± SEM, * *p* < 0.05.

ROS generation under normal conditions is relatively low, however, ROS levels can profoundly increase during conditions of cell stress, such as reperfusion following ischemia [25]. Given the previously reported loss of S47 phosphorylation of Cyt*c* under ischemia, we assessed the role of this modification using an oxygen-glucose deprivation/reoxygenation (OGD/R) model. This system was used to model ischemia/reperfusion injury in cultured cells [26]. We exposed our stably transfected cell lines to 1% oxygen (simulated hypoxia/ischemia) in glucose-depleted media for 90 min, followed by reperfusion with MitoSOX supplemented complete culture media for 30 min. MitoSOX fluorescence of the WT unphosphorylated Cyt*c* expressing cells doubled upon reperfusion. In striking contrast, MitoSOX fluorescence in the S47E cell line and S47A cell line showed a non-significant increase of 8.8% and 9.2%, respectively (Figure 3A). The S47E cell line displayed significantly reduced ROS levels under control conditions. However, upon exposure to simulated ischemia/reperfusion, this effect was even more prominent with a doubling of MitoSOX fluorescence in the WT cell line. This result depicts a protective role for the S47E phosphomimetic replacement in the context of ischemia/reperfusion injury. Similarly, we measured ΔΨ_m_ upon simulated reperfusion using the same OGD/R model. Here, we captured ΔΨ_m_ upon reperfusion. Similar to baseline conditions, the WT cell line showed a significantly higher ΔΨ_m_ compared to the S47E cell line upon reperfusion, indicating that phosphomimetic substitution maintained lower ΔΨ_m_ levels (Figure 3B). Our data thus drew a direct relationship between the speed of the electron transfer between Cyt*c* and COX, intact cell respiration, ΔΨ_m_, and ROS.

### 3.5. Phosphomimetic S47E Cytc Protects against Cell Death upon OGD/R and H_2_O_2_ Treatment

**Figure 4:**
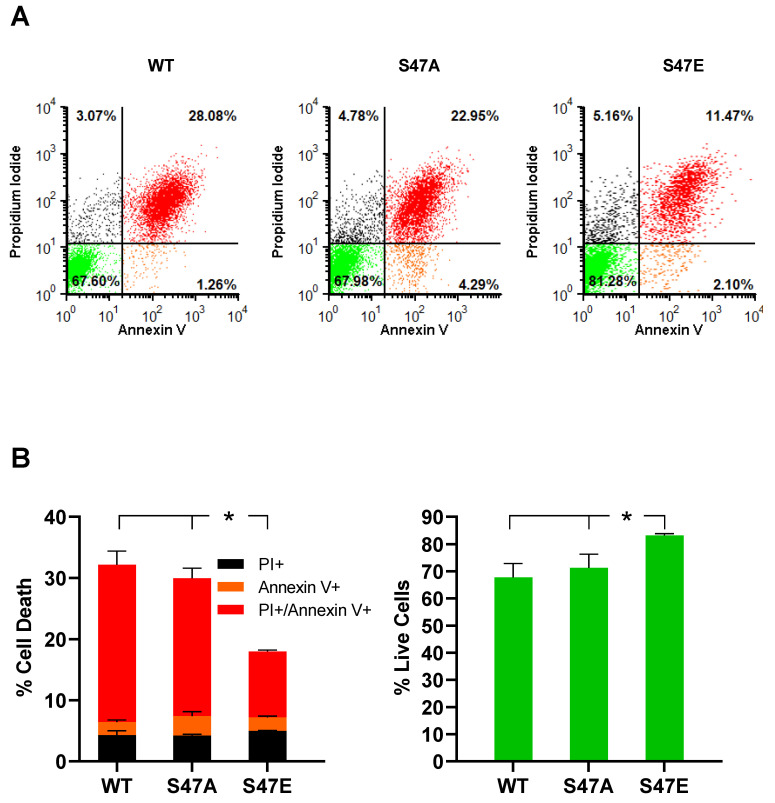
(**A**) Flow cytometry scatter plots representing necrotic (black, positive only for propidium iodide), early apoptotic (orange, positive only for annexin V), late apoptotic (red, double-positive for propidium iodide and annexin V), and live cells (green, unstained) in WT, S47A, S47E Cyt*c* expressing cells following exposure to oxygen-glucose deprivation for 12 h, followed by reoxygenation for 1 h. (**B**) Cell death represented as fractions of necrotic (black), early apoptotic (orange), and late apoptotic (red) cells. Live cells, represented in the lower left quadrant in the flow cytometry scatter plots, are shown as percent of total cells. The empty vector cell line was excluded due to near-complete cell death in the absence of glucose (*n* = 3–4). Data are represented as means ± SEM, * *p* < 0.05.

**Figure 5:**
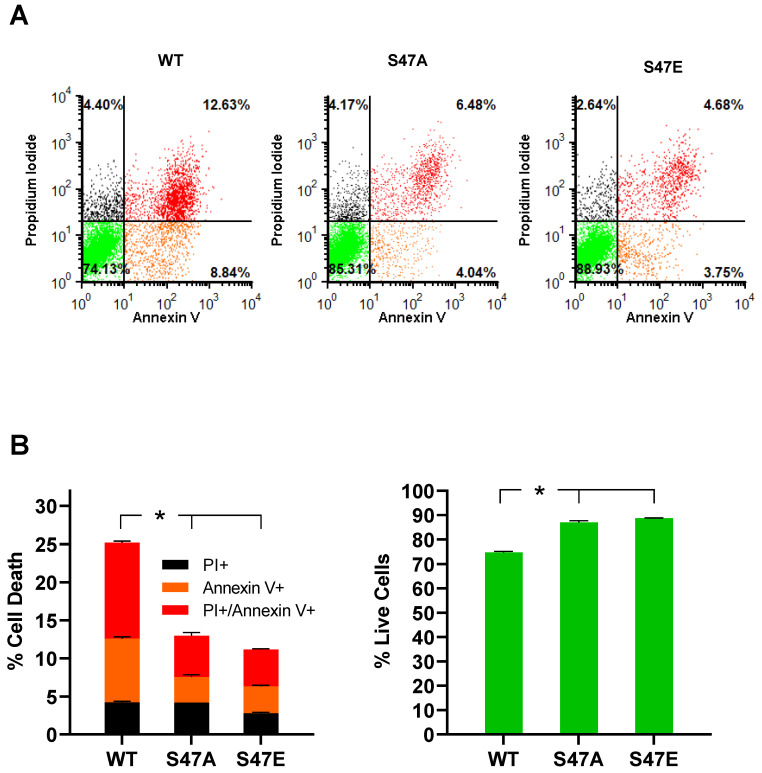
(**A**) Flow cytometry scatter plots representing necrotic (black, positive only for propidium iodide), early apoptotic (orange, positive only for annexin V), late apoptotic (red, double-positive for propidium iodide and annexin V), and live cells (green, unstained) in WT, S47A, S47E Cyt*c* expressing cells after treatment with 400 µM H_2_O_2_ for 16 h. (**B**) Cell death represented as fractions of necrotic (black), early apoptotic (orange), and late apoptotic (red) cells. Live cells, represented in the lower left quadrant in the flow cytometry scatter plots, are shown as percent of total cells (*n* = 3). Data are represented by means ± SEM, * *p* < 0.05.

We assessed the impact of OGD/R on cell death using annexin V/propidium iodide (PI) staining, followed by flow cytometry analysis. Phosphatidylserine (PS) is a membrane lipid that is normally present in the inner leaflet of the plasma membrane. However, during apoptosis, PS flips to the outer leaflet of the plasma membrane. Annexin V, a calcium-dependent protein that binds to PS when fluorescently labeled, can be used to detect PS that is exposed on the outside of apoptotic cells. Apoptotic cells can be distinguished from necrotic cells by co-staining with PI [27]. PI is not cell-permeable and stains for nuclear DNA. Therefore, PI only stains necrotic cells, in which the cell membrane is damaged [28]. As a result, annexin V-positive cells represent early apoptotic cells, PI-positive cells represent necrotic cells, and annexin V- and PI-positive cells represent late apoptotic cells. Annexin V/PI staining of our cell lines following 12 h of OGD and 1 h of reperfusion showed that cells expressing S47E phosphomimetic Cyt*c* resulted in significantly lower cell death (18%) compared to the WT and S47A cell lines that resulted in 32.3% and 30% cell death, respectively (Figure 4A,B). We further assessed cell death in these cell lines upon H_2_O_2_ treatment, which causes cell death by inducing oxidative stress [29]. Annexin V/PI staining after treatment with 400 µM H_2_O_2_ for 16 h showed high levels of cell death in the WT control cells (25.2%), whereas cells expressing S47E Cyt*c* and S47A Cyt*c* were better protected with only 11.2% and 13% cell death, respectively (Figure 5A,B). The protection of the S47A cell line against cell death upon H_2_O_2_ treatment, in comparison to OGD/R exposure, might be attributed to higher capacity of H_2_O_2_ scavenging ability shown by S47A recombinant Cyt*c* (Figure S3B).

### 3.6. Akt Is a Potential Kinase Mediating S47 Phosphorylation of Cytc

**Figure 6:**
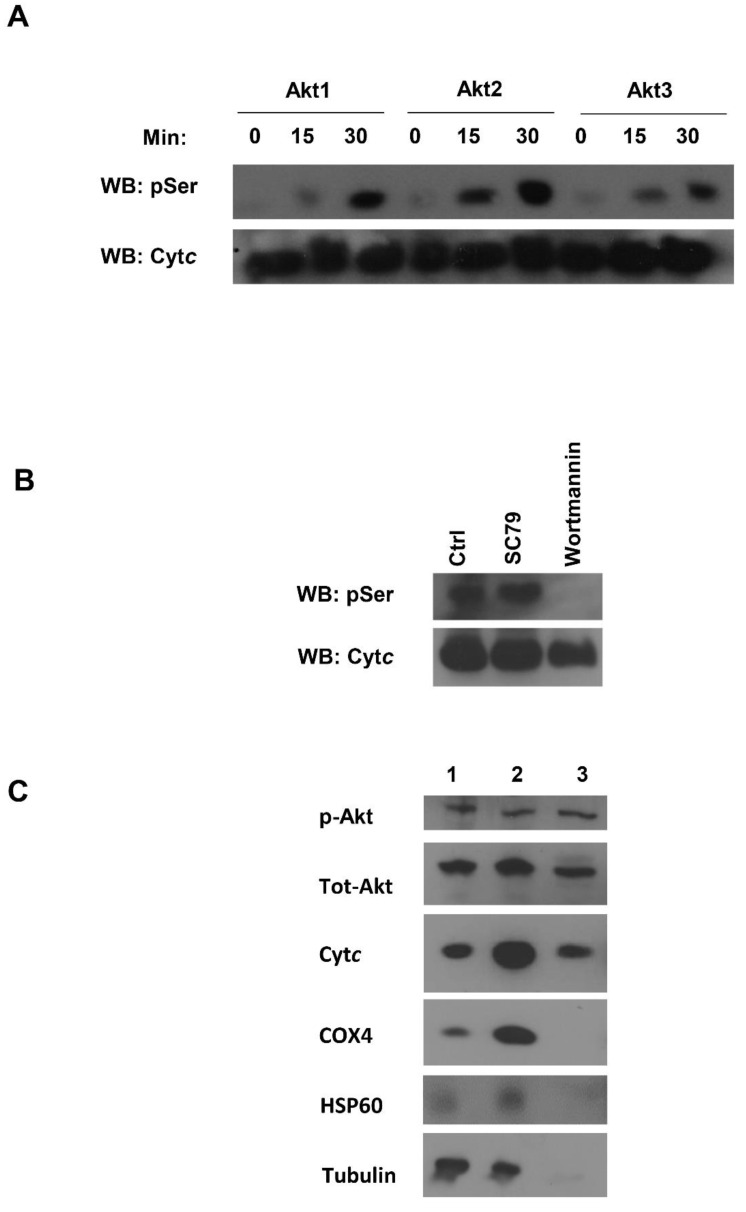
(**A**) In vitro Akt kinase assay showing a time-dependent increase in serine phosphorylation at 0, 15, and 30 min. BSA was used as a negative control (not shown). (**B**) The purified pig brain Cyt*c,* following SC79 (Akt activator), wortmannin (Akt inhibitor), and control (DMSO) treatments, was assessed for S47 phosphorylation. (**C**) Pig brain mitochondria fractionation. Lane1, pig brain tissue lysate; lane 2, crude mitochondria; lane 3, mitochondrial intermembrane space (IMS) fraction. COX4, HSP60, and tubulin were used as an inner mitochondrial membrane (IMM), mitochondrial matrix, and cytosolic markers, respectively.

Our next goal was to determine the potential kinase that targets this highly regulatory S47 residue of Cyt*c* for phosphorylation. We performed an in silico analysis using NetPhos 3.1 software [30] and found that Akt kinase could potentially target Cyt*c*. We assessed the likelihood of S47 phosphorylation by performing an in vitro kinase assay with all three isoforms of Akt (Akt 1, Akt 2, and Akt 3). S47 is the only serine residue present in rodent Cyt*c*. After probing the Akt kinase reactions with a phosphoserine antibody, we observed that all three isoforms of Akt could phosphorylate Cyt*c* in a time-dependent manner in vitro (Figure 6A). In order to determine if the S47 phosphorylation of Cyt*c* is mediated by Akt in tissues, we treated pig brain homogenates with a pharmacological activator of Akt, SC79, and an inhibitor of Akt, wortmannin. Akt activator SC79 binds to the pleckstrin homology (PH) domain of Akt and changes the conformation that makes it favorable for phosphorylation by upstream kinases [31]. Wortmannin acts through non-competitive, irreversible inhibition of PI3K at a concentration of 2-4 nM [32]. Wortmannin has been found to inhibit Akt phosphorylation in a time and dose-dependent manner [33]. It inhibits myosin light chain kinase (MLCK) at a much higher concentration of 0.2 µM and is not considered to be an inhibitor of phosphatidylinositol-4-kinase, protein kinase C, or c-Src tyrosine kinase [32]. Post-treatment, Cyt*c* was purified from these tissue samples and assessed for its serine phosphorylation state. In the control sample, there was a basal level of serine phosphorylation. Upon treatment with the Akt inhibitor wortmannin, complete dephosphorylation of Cyt*c* was observed, suggesting that this phosphorylation might be mediated by Akt. Upon treatment with the Akt activator SC79, serine phosphorylation of Cyt*c* was observed (Figure 6B). Akt is known to translocate to the mitochondria when this signaling pathway is active, including the mitochondrial intermembrane space (IMS) where Cyt*c* is located. This has been shown in several cell systems, including brain cells [34,35,36]. It has been further shown that of the three Akt isoforms, Akt2 localizes to the mitochondria [37,38]. We confirmed Akt translocation to the mitochondrial IMS in our pig brain mitochondrial fractions. Our mitochondrial fractionation results showed that S473-phosphorylated Akt and pan-Akt translocated to the mitochondrial IMS. Tubulin was used as a cytosolic marker, while COX4 and HSP60 were used as inner mitochondrial membrane and mitochondrial matrix markers, respectively (Figure 6C).

## 4. Discussion

Our lab previously showed that S47 phosphorylation of Cyt*c* is an important modification that regulates COX and caspase-3 activity in the mammalian brain [12]. In this paper we expanded on our studies by characterizing phosphomimetic S47E Cyt*c* in a cell culture system. We previously established that S47E is an excellent mimic to study S47 phosphorylation of Cyt*c* based on the spatial arrangement of S47E in the crystal structure (6N1O.pdb) and functional studies using in vivo S47 phosphorylated and S47E Cyt*c* side by side [12]. We overexpressed WT, S47A, and S47E Cyt*c* in Cyt*c* double-knockout lung fibroblasts [21]. It is important that both the somatic and testes isoforms of Cyt*c* are knocked out from these cells because, in the absence of the somatic isoform, the testes isoform may be induced [21]. Both somatic and testes Cyt*c* encode Cyt*c* isoforms consisting of 104 amino acids, with 86% identical residues between the two [39]. There are multiple pseudogenes of somatic Cyt*c* in human and mouse genomes [40]. However, humans express only one active isoform of Cyt*c* that shows features of both the rodent somatic and testes isoforms. Once Cyt*c* is stably reintroduced into the Cyt*c* double-knockout cells, they start expressing higher levels of complexes I, III, and IV, which are known to form supercomplexes and are downregulated in the absence of Cyt*c* [41,42]. This supports the findings from a previous study that suggested a role of Cyt*c* in the assembly and stability of respiratory supercomplexes [43].

The effect on intact cell respiration observed in the presence of S47E Cyt*c* was similar to the decrease in COX activity reported in vitro with a Clark-type oxygen electrode [12] (Table 1). The observation that the rate of the ETC flux could be modulated by modifications of Cyt*c* further supported our model that the reaction between Cyt*c* and COX can serve as the rate-limiting step of the ETC controlling overall ETC flux [2]. In addition, there is evidence that the S47 residue of Cyt*c* may also interact with the *bc*_1_ complex [44]. Therefore, modifications at this residue may affect electron transfer from the *bc*_1_ complex, lowering overall ETC activity. The phosphomimetic S47E cell line showed higher glycolytic flux compared to the unphosphorylated WT cell line, likely compensating for the lower mitochondrial oxygen consumption. The ATP level for S47E was lower than that of WT and S47A; however, the difference was not statistically significant. It should also be noted that in vivo phosphorylation of the Cyt*c* pool in the brain will never reach 100%, whereas all Cyt*c* in the cells expressing S47E Cyt*c* possess the phosphomimetic modification. Therefore, the effect of Cyt*c* phosphorylation on ATP levels may be negligible in the mammalian brain.

The partial inhibition of respiration observed in the S47E Cyt*c* expressing cell line is important because, under physiological conditions, respiration and mitochondrial ΔΨ_m_ are directly related. In addition, it has been estimated that 95% of ROS are generated from electron leak in the respiratory complexes of the ETC in normal cells [25]. Maintenance of intermediate ΔΨ_m_ (100–120 mV) avoids excessive ROS generation but provides the full capability to produce ATP because maximal rates of ATP synthesis by ATP synthase take place at an intermediate ΔΨ_m_ of 100–120 mV [45]. At ΔΨ_m_ > 140 mV, ROS production at ETC complexes I and III increases exponentially, whereas mitochondria of resting cells with an optimal, intermediate ΔΨ_m_ do not produce significant amounts of ROS [46,47]. This relationship between ΔΨ_m_ and ROS is evident from our results and further tested in the context of ischemia/reperfusion injury by using an OGD/R cell culture model. The unphosphorylated WT Cyt*c* expressing cells showed a significant increase in ROS following OGD/R. The phosphomimetic cell line attenuated ROS production, further establishing a protective role for S47 phosphorylation in the context of ischemia/reperfusion injury. Our model proposes that cell signaling pathways target the ETC. However, under conditions of stress, such as ischemia/reperfusion, there is an influx of calcium into the mitochondria [48,49,50]. It has been demonstrated that calcium is a potent activator of the mitochondrial ETC [51]. This hyperactivity is likely mediated by dephosphorylation of mitochondrial proteins, including Cyt*c*, mediated by calcium-dependent phosphatases [52]. The increase in ETC activity leads to pathologically high ΔΨ_m_ levels and a burst of ROS that triggers cell death as a result of ischemia/reperfusion injury (reviewed in [5]). As predicted from our model, the protective role of S47E Cyt*c* was evident from significantly lower cell death reported upon exposure to OGD/R. In addition, the phosphomimetic cell line showed greater cell viability upon treatment with hydrogen peroxide. Other contributing factors explaining reduced cell death could be lowered caspase-3 activity and cardiolipin peroxidase activity as was previously reported for S47 phosphomimetic Cyt*c* [12,53]. S47A and S47E Cyt*c* resulted in 38% and 35% caspase-3 activity, respectively, compared to WT. Both the mutants also resulted in significantly lower cardiolipin peroxidase activity compared to WT at varying ratios of cardiolipin to Cyt*c* (Table 1). These functional parameters may explain the lower cell death observed when S47 residue of Cyt*c* is modified.

Finally, we presented data suggesting that pro-survival kinase Akt could target S47 for phosphorylation. S47 is the only serine residue present in rodent Cyt*c*, making it a unique regulatory site in mammals. In vitro kinase assays with all three Akt isoforms resulted in a time-dependent increase in serine phosphorylation. We also used pharmacological Akt activator SC79 and inhibitor wortmannin in pig brain tissue lysates to modulate the signaling pathway. The analysis of Cyt*c* phosphorylation state post-treatment resulted in complete dephosphorylation of Cyt*c* in the presence of wortmannin, suggesting a potential role for Akt in mediating this pathway. Interestingly, several studies have shown that Akt activator SC79 is an effective treatment for cerebral ischemia/reperfusion injury in animal models [54,55,56]. We and others have shown that activated, phosphorylated Akt translocates to the mitochondrial IMS where Cyt*c* resides [34,35,36]. This suggests that Cyt*c* is targeted for S47 phosphorylation by Akt, which, in turn, results in optimal, intermediate ΔΨ_m_ levels, lower ROS production, and significant protection from cell death upon OGD/R and other stresses. This experimental model is further supported by our recently discovered specific wavelengths of infrared light that partially inhibits the reaction between Cyt*c* and COX, mimicking a protective effect similar to that observed with S47 phosphorylation of Cyt*c* [57,58]. In conclusion, our study establishes the cytoprotective role of S47 phosphorylation in cerebral ischemia/reperfusion injury.

## Figures and Tables

**Table 1:** Summary of the effect of WT, S47A, and S47E Cyt*c* on different mitochondrial functions involving Cyt*c*.

Cyt*c* Function	WT	S47A	S47E/S47D	EV	Reference
Brain cytochrome *c* oxidase (COX) activity	100%	~82%	~46%	-	[12]
Caspase-3 activity	100%	~38%	~35%	-	[12]
	100%	~50%	~30%		[53]
Cardiolipin peroxidase activity (cardiolipin: Cyt*c*)	100% (10:1)	~37% (10:1)	~50% (10:1)	-	[12]
	100% (20:1)	~54% (20:1)	~51% (20:1)		
	100% (100:1)	~60% (100:1)	~80% (100:1)		[53]
Intact cell respiration	100%	~81%	~42%	~15%	This study
Mitochondrial membrane potential	100%	~93%	~79%	~55%	This study
Mitochondrial ROS production	100%	~74%	~70%	~63%	This study
ATP production	100%	~100%	~84%	~28%	This study
Cell death (after OGD/R)	~32%	~30%	~18%	-	This study
Cell death (after H_2_O_2_ treatment)	~25%	~13%	~11%	-	This study

## Supplementary Materials

The following are available online at https://www.mdpi.com/2073-4409/9/8/1843/s1, Figure S1: Mitochondrial stress test in Seahorse media supplemented with 10 mM galactose, Figure S2: Mitochondrial stress test in Seahorse media supplemented with 10 mM glucose and 10 mM sodium pyruvate, Figure S3: Glycolysis stress test based on extracellular acidification rate (ECAR) of cells in Seahorse media supplemented with 10 mM glucose and 10 mM sodium pyruvate, and rate of Cyt*c* oxidation upon addition of H_2_O_2_.

## Abbreviations

COX	cytochrome *c* oxidase
Cyt*c*	cytochrome *c*
ΔΨ_m_	mitochondrial membrane potential
ETC	electron transport chain
OGD/R	oxygen-glucose deprivation/reoxygenation
OxPhos	oxidative phosphorylation
ROS	reactive oxygen species
