# Supplementary material for: Brain-Specific Serine-47 Modification of Cytochrome c Regulates Cytochrome c Oxidase Activity Attenuating ROS Production and Cell Death: Implications for Ischemia/Reperfusion Injury and Akt Signaling
Source: Cells. 2020 Aug 6;9(8):1843. doi: 10.3390/cells9081843 (PMC7465522; doi:10.3390/cells9081843)
Supplement: Supplementary file 1 [file cells-09-01843-s001.pdf]

Supplementary Materials

Brain-Specific Serine-47 Modification of Cytochrome *c* Regulates Cytochrome *c* Oxidase Activity Attenuating ROS Production and Cell Death: Implications for Ischemia/Reperfusion Injury and Akt Signaling

Hasini A. Kalpage [1], Junmei Wan [1], Paul T. Morse [1], Icksoo Lee [2], Maik Hüttemann [1,3,*]

1 Center for Molecular Medicine and Genetics, Wayne State University, Detroit, MI 48201, USA
[2] College of Medicine, Dankook University, Cheonan-si, Chungcheongnam-do 31116, Republic of Korea
[3] Department of Biochemistry, Microbiology and Immunology, Wayne State University, Detroit, MI 48201, USA
[*] Correspondence: mhuttema@med.wayne.edu; Tel.: (+1) 313-577-9150; Fax: (+1) 313-577-5218.

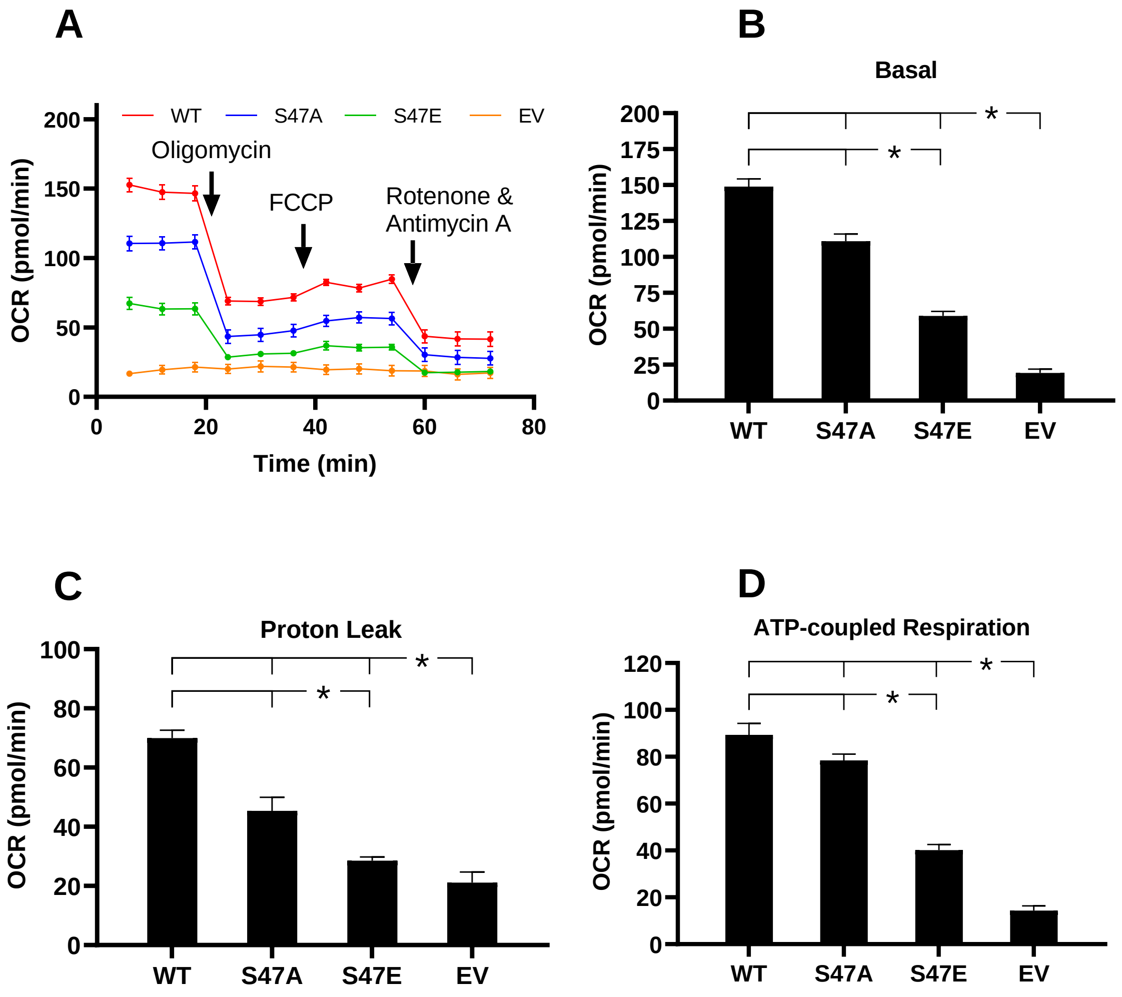

Figure S1. (**A**) Mitochondrial stress test of cells stably expressing EV and WT, S47A, S47E Cytc in Seahorse media supplemented with 10 mM galactose (n = 5–6). FCCP addition does not result in the expected maximal respiration. (**B**) Basal mitochondrial respiration. (**C**) Proton leak (basal respiration not coupled to ATP production after addition of oligomycin). (**D**) ATP-coupled respiration in Seahorse media supplemented with 10 mM galactose. Data are represented as means ± SEM, * $p < 0.05$.

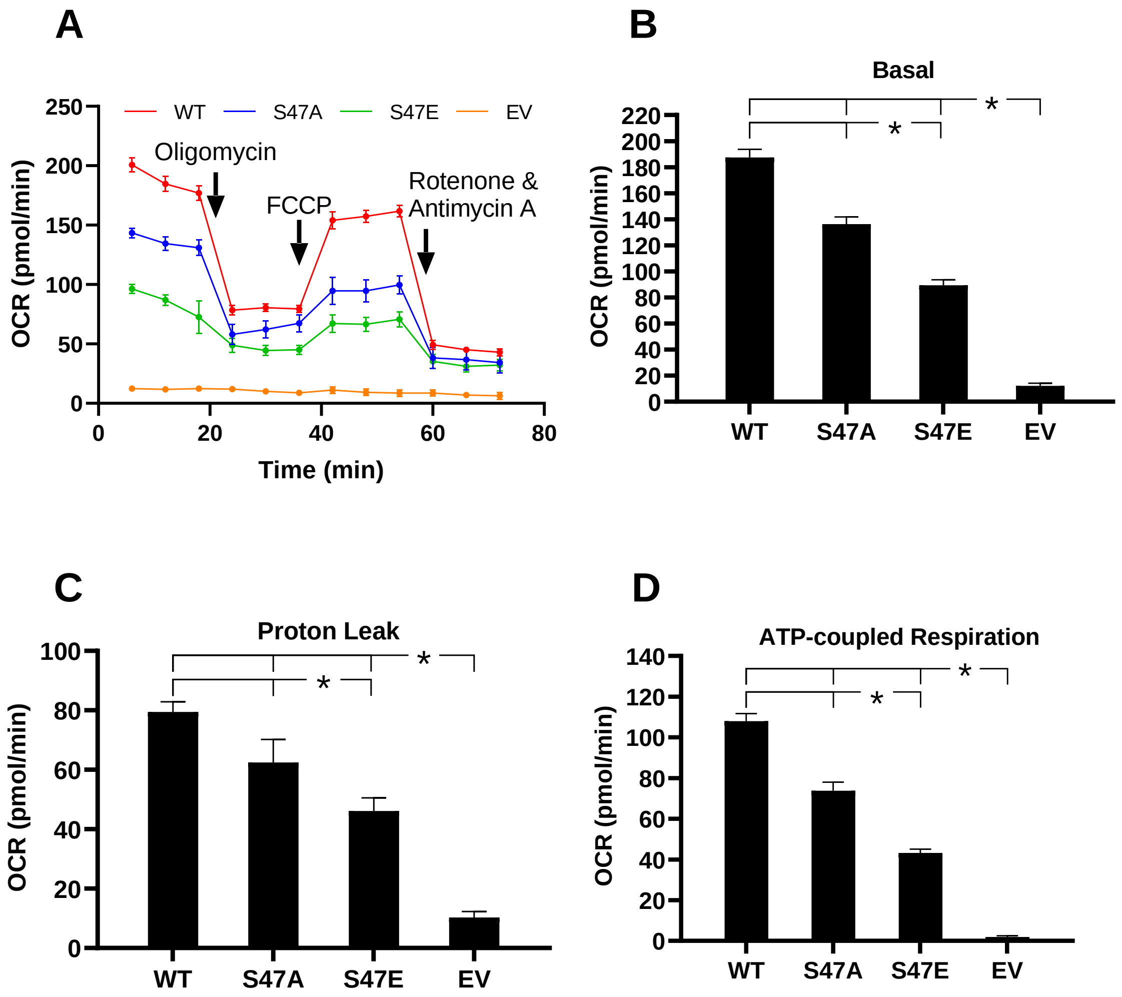

Figure S2. (**A**) Mitochondrial stress test of cells stably expressing EV and WT, S47A, S47E Cytc in Seahorse media supplemented with 10 mM glucose and 10 mM sodium pyruvate (n = 5–6). FCCP addition does not result in the expected increased respiration suggesting that the compound is toxic to lung embryonic fibroblast cells. (**B**) Basal mitochondrial respiration. (**C**) Proton leak (basal respiration not coupled to ATP production after addition of oligomycin). (**D**) ATP-coupled respiration in Seahorse media supplemented with 10 mM galactose. Data are represented as means ± SEM, * $p < 0.05$.

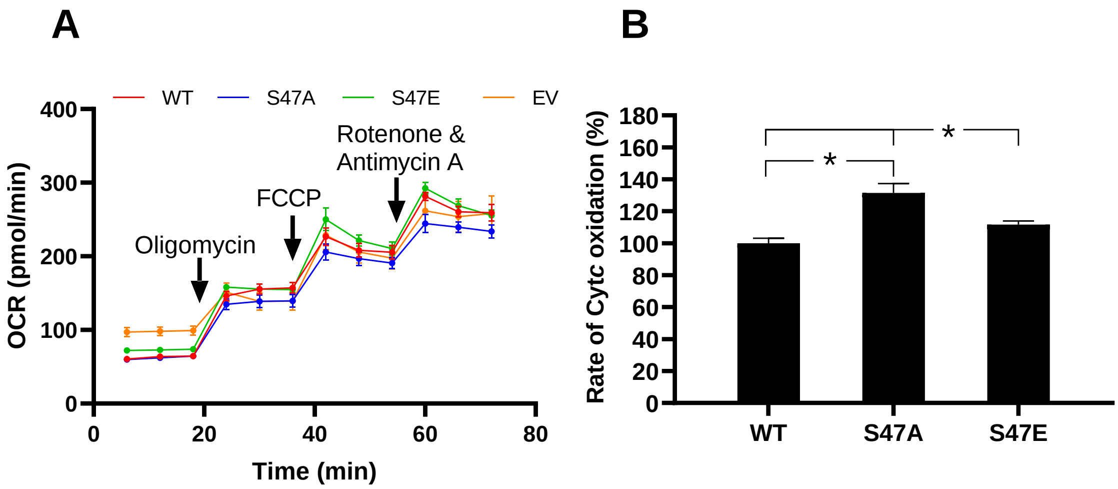

Figure S3. (**A**) Glycolysis stress test based on extracellular acidification rate (ECAR) of cells stably expressing EV and WT, S47A, S47E Cyt*c* in Seahorse media supplemented with 10 mM glucose and 10 mM sodium pyruvate (n = 4–6). (**B**) Rate of Cyt*c* oxidation upon addition of 100 µM H_2O_2 (n = 3). Data are represented as means ± SEM, * $p < 0.05$.
